# Postural Responses to Sudden Horizontal Perturbations in Tai Chi Practitioners

**DOI:** 10.3390/ijerph18052692

**Published:** 2021-03-07

**Authors:** Jernej Sever, Jan Babič, Žiga Kozinc, Nejc Šarabon

**Affiliations:** 1 Center Premik, Ltd., Center for Psychophysical Development, 1000 Ljubljana, Slovenia; jernej_sever@yahoo.com; 2 Laboratory for Neuromechanics and Biorobotics, Jožef Stefan Institute, 1000 Ljubljana, Slovenia; jan.babic@ijs.si; 3 Andrej Marusic Institute, Department of Health Study, University of Primorska, 6000 Koper, Slovenia; ziga.kozinc@fvz.upr.si; 4 Faculty of Health Sciences, University of Primorska, 6310 Izola, Slovenia; 5 Human Health Department, InnoRenew CoE, 6310 Izola, Slovenia; 6 Laboratory for Motor Control and Motor Behavior, S2P, Science to Practice, Ltd., 1000 Ljubljana, Slovenia

**Keywords:** martial arts, balance, postural control, reaction, posture

## Abstract

Tai Chi has been shown to elicit numerous positive effects on health and well-being. In this study, we examined reactive postural control after sudden unloading horizontal perturbations, which resembled situations encountered during Tai Chi. The study involved 20 participants, 10 in the Tai Chi group (age: 37.4 ± 7.8 years), who had been regularly training the push-hand technique for at least 7 years, and 10 in the control group, consisting of healthy adults (age: 28.8 ± 5.0). Perturbations were applied at three different positions (hips, shoulders, and arms) via the load-release paradigm. Twenty measurements were carried out for each perturbation position. We measured peak vertical and horizontal forces on the ground (expressed percentage of body mass (%BM)), peak center of pressure displacement and peak horizontal and vertical velocities at the knee, hip and shoulder joints. The Tai Chi group exhibited smaller increases in vertical ground reaction forces when perturbations were applied at the hips (11.5 ± 2.1 vs. 19.6 ± 5.5 %BW; *p* = 0.002) and the arms (14.1 ± 4.2 vs. 23.2 ± 8.4 %BW; *p* = 0.005). They also responded with higher horizontal force increase after hip perturbation (16.2 ± 3.2 vs. 13.1 ± 2.5 %BW; *p* < 0.001). Similar findings were found when observing various outcomes related to velocities of vertical movement. The Tai Chi group also showed lower speeds of backward movement of the knee (*p* = 0.005–0.009) after hip (0.49 ± 0.13 vs. 0.85 ± 0.14 m/s; *p* = 0.005) and arm perturbations (0.97 ± 0.18 vs. 1.71 ± 0.29 m/s; *p* = 0.005). Center of pressure displacements were similar between groups. Our study demonstrated that engaging in Tai Chi could be beneficial to reactive postural responses after sudden perturbations in a horizontal direction; however, future interventional studies are needed to directly confirm this. Moreover, because of the age difference between the groups, some confounding effects of age cannot be ruled out.

## 1. Introduction

Tai Chi is a Chinese martial art, primarily known in the West as a stand-alone version of moving meditation. It includes slow and controlled movements, usually preformed through a predefined exercise sequence known as a Tai Chi form. Previous studies have reported that practicing Tai Chi can improve flexibility and postural control during standing [1,2] and walking [3]. Importantly, it also improved the ability to recover balance after a slip in the elderly, which is crucial for prevention of falls [3]. Furthermore, several studies have investigated the clinical utility of Tai Chi exercise. For instance, a meta-analysis indicated that Tai Chi exercise can help reduce motion-related pain and stiffness in osteoarthritis patients [4]. Tai Chi can also be a safe and effective complementary exercise in Parkinson’s disease [5,6] and has been reported as a feasible approach to improve general physical fitness in patients with obstructive pulmonary disease [7]. While Tai Chi (similar to other martial arts [8]) is consistently shown to have beneficial health effects, some of the underlying mechanisms of its effects, especially those related to postural control, remain somewhat unknown. Tai Chi movements are underlain with precise neuromuscular control of the posture, combining mobility and stability [9]. Regular practice of Tai Chi may improve the range of motion of joints in the lower extremities, especially in the ankle [9,10]. Biomechanical studies have shown improved stability in single-leg stance [11] and more efficient preparation of cued steps [12].

Unlike many martial arts, Tai Chi is not characterized by fighting elements such as strikes or kicks [8]. Tai Chi movements involve pushing, pulling, and redirecting the opponent (also termed the “push-hand technique”) in order to manipulate his/her center of mass (CoM) in relation to the base of support. A case study of a Tai Chi teacher revealed a controlled shift of body weight from front to back foot when an inexperienced participant tried to push him out of balance. Such shift in body weight distribution are paramount to maintaining postural stability [13]. During Tai Chi duels, the changes in external forces (i.e., the opponent pushing, pulling, and releasing) occur with varying intensity, timing and direction. To maintain postural control, the body must respond quickly and effectively to these changes.

While improved static postural control [11] and improved gait pattern [12] have been shown in Tai Chi practitioners, reactive postural control in response to sudden surface translations was not substantially improved after 4 weeks of Tai Chi training [14]. Indeed, using surface translations that quickly induce an offset between CoM and base of support is a common paradigm to explore reactive postural balance [15,16]. However, this type of perturbation is not typical in martial arts or other contact sports. In Tai Chi, large pushing or pulling forces are typically experienced at the height of CoM and above. Because adaptations to stability training are highly task-specific [17], it could be expected that reactive postural responses to external perturbations at this level are improved with Tai Chi training. Applying sudden external perturbations (by suddenly applying or releasing a load) to the upper body has been also extensively used to study postural control [18,19,20,21,22]; however, to the best of our knowledge, no study to date has examined reactive postural responses to external upper body perturbations in Tai Chi practitioners.

In this study, we investigated the reactive postural control to upper body perturbations in Tai Chi practitioners. Previous experiments [22,23] demonstrated that the height (shoulder vs. hip) of the application of the unloading perturbation significantly affects the postural responses. Specifically, it was shown that perturbation higher up the body induced larger changes in trunk inclination, internal moments and back muscle activity, compared to the perturbation applied lower [22,23]. In view of these reports (and in line with the observations of task-specific improvements after stability training [17]), it could be that the adaptations in Tai Chi practitioners are more evident when perturbations are applied at a body site that typically serves as contact point between the opponents during a Tai Chi fight using the push-hand technique (arms and hips) compared to the situations when perturbation are applied at the shoulder. Therefore, our aim was to compare Tai Chi practitioners and comparable control subjects in terms of reactive postural control to unloading perturbations, applied at different body sites in situations that are similar to those that participants have to cope with in push-hands conditions. The examined parameters included the magnitudes of horizontal and vertical ground reaction forces, the amplitudes and velocities of center of pressure displacement and kinematic velocities of body movement at different heights. These variables were tracked after unloading perturbations applied to hips, shoulders and arms. We hypothesized that the Tai Chi practitioners will exhibit superior postural control, reflected in lower ground reaction forces, as well as the amplitudes and velocities of center of pressure displacement and kinematic velocities of body movement.

## 2. Materials and Methods

### 2.1. Participants

The study involved 10 healthy Tai Chi participants who regularly performed the push-hand technique (according to Yang School of Tai Chi) and 10 healthy and physically active participants that served as the control group. To be included in the former group, the participants had to have participated in Tai Chi practice for at least 1 year. The actual experience with Tai Chi of the final sample was 7.0 ± 5.5 years, and all participants reported performing the trainings 3–5 times per week under the supervision of a Tai Chi instructor. The control group consisted of participants with no Tai Chi training history; however, they had been participating various recreational physical activities at a comparable weekly frequency. The experimental protocol was approved by the Research Ethics Committee at the Faculty of Arts, University of Ljubljana, and at the National Medical Ethic Committee (approval code: 112/06/13). The study protocol was consistent with the Oviedo Convention and the Declaration of Helsinki as it relates to human research. All participants signed a consent form before participation.

### 2.2. Tasks and Equipment

The participant first performed a 10-min warm-up consisting of: in-place running (~4 min), dynamic stretching the main muscle groups (~3 min), and short bouts of activation strength exercises (~3 min).

**Figure 1:**
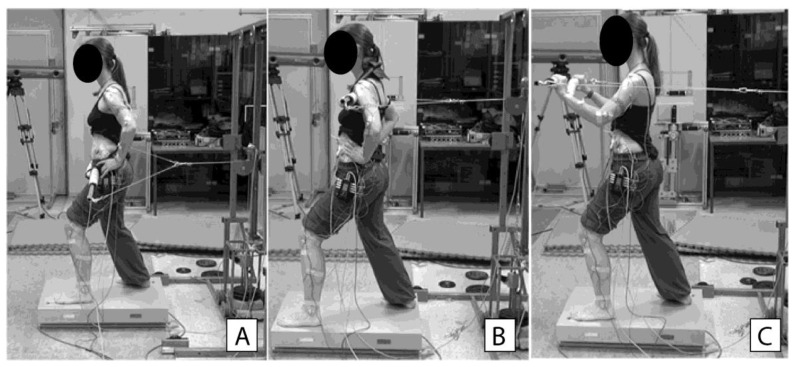
Experimental setup. The padded pole to load the participant’s body at the height of the hips (**A**), shoulders (**B**) and through the arms (**C**).

The length and width of the step were determined as 60 and 40 % of the height of the 5th lumbar vertebrae, respectively, which resembled a typical posture for Tai Chi forms (Figure 1). A cable pulley set-up for application and sudden release of horizontal force was created. The load was attached to a steel wire through a magnetic trigger, which allowed random and sudden release. The steel wire was attached to a padded pole, which was used to apply horizontal force on the participant’s body in different positions (Figure 1).

The perturbations were applied on the trunk at hip height (aligned with greater trochanters) (Figure 1A) and shoulder height (the pole placed at upper sternum; Figure 1B) and through the arms (Figure 1C). For the latter, the participants held the pole at the height of sternum with elbows bent to 90°. When the participant was ready, he/she lifted the load with a short movement forward and waited for a sudden release. The release was triggered randomly between 3 and 7 s after the participant assumed the starting position.

### 2.3. Data Acquisition and Analysis

Data were collected with two synchronized systems: (1) a bilateral force plate (9281CA, Kistler, Instrument AG, Winterthur, Switzerland) and (2) a 3D motion analysis system (Optotrak 3D Investigator, Northern Digital Inc., Waterloo, ON, Canada) with two optical modules. The kinetic and kinematic data were sampled with 1000 and 100 Hz, respectively. Ground reaction force data were low pass filtered (Butterworth, 2nd order, 10 Hz). Kinematic sensors were attached at one side of the body at the knee (lateral condyle), hip (spina illiaca anterior superior), and shoulder (acromion). In terms of kinetic variables, we calculated peak vertical and horizontal ground reaction forces. The following outcome measures were analyzed: (a) peak horizontal (F_y_) and vertical (F_z_) ground reaction forces (expressed as the difference between the peak and baseline (preperturbation) levels); (b) peak displacement of center of pressure (CoP); (c) peak horizontal (V_y_) and vertical (V_z_) velocities of movement at the knee, hip and shoulder. In all conditions, 2 familiarization repetitions were performed, after which 20 repetitions were recorded. The break between repetitions was set at 1 min. For all of the outcomes, the average of all repetitions was taken for further analyses.

### 2.4. Statistical Analyses

Statistical analysis was performed using SPSS 25 (SPSS inc, Chicago, IL, USA). Descriptive statistics are reported as mean ± standard deviation. Mixed model analysis of variance was used for the main analysis, with the groups (Tai Chi, control) as the between-participant factor and perturbation location (hip, shoulder, arms) as the within-participant factor. Mauchly’s test was used to assess sphericity of both groups and the Greenhouse–Geiser correction was applied when needed. Statistically significant differences between the main effect of group were further investigated with separate post-hoc t-tests with Bonferroni correction for each perturbation area, and the effect sizes were expressed as Cohen’s d [24] and interpreted as such trivial (>0.2), small (0.2–0.5) medium (0.5–0.8), large (0.8–1.2) and very large (>1.2). The statistical significance threshold was set to 0.05. According to a study by Wolf et al. [10], small to moderate (Cohen’s d = 0.25–0.5) improvements in postural balance are expected after Tai Chi training. Thus, considering the lower end of this range (0.25) as the expected effect size, we calculated the required sample size to be 18 in order to achieve statistical power of 80 % at alpha set at 0.05 (GPower software, version 3.0.10, Düsseldorf, Germany).

## 3. Results

The basic demographics data for both groups is presented in Table 1.

**Figure 2:**
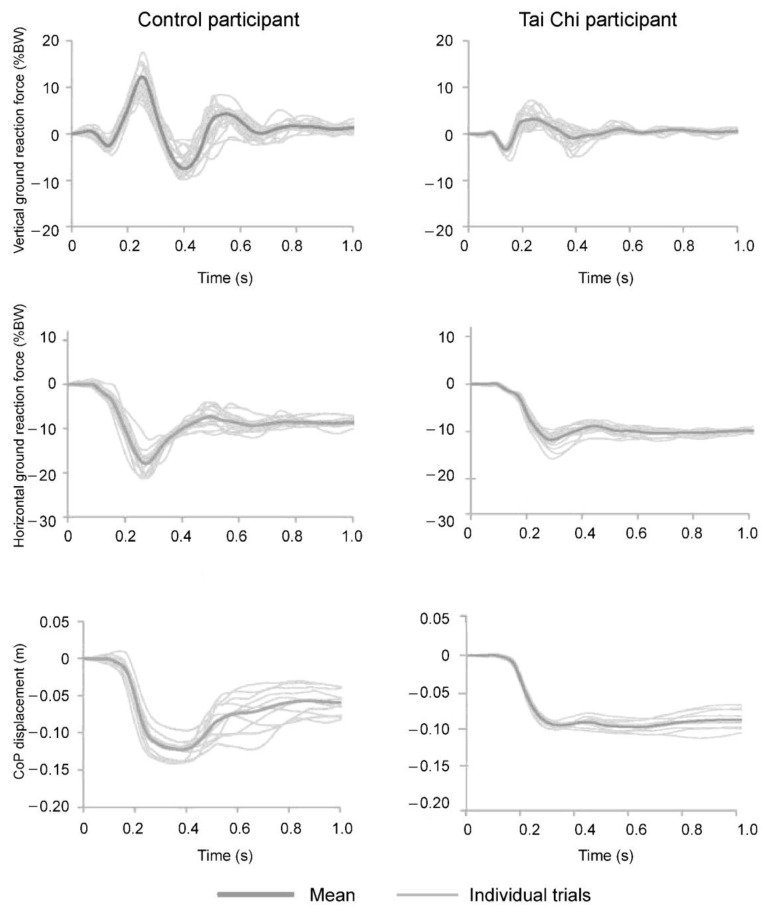
Results of two representative subjects from the control group (**left**) and Tai Chi (**right**) group for responses in vertical (**top**) and horizontal (**middle**) ground reaction forces and center of pressure displacement (**bottom**).

**Figure 3:**
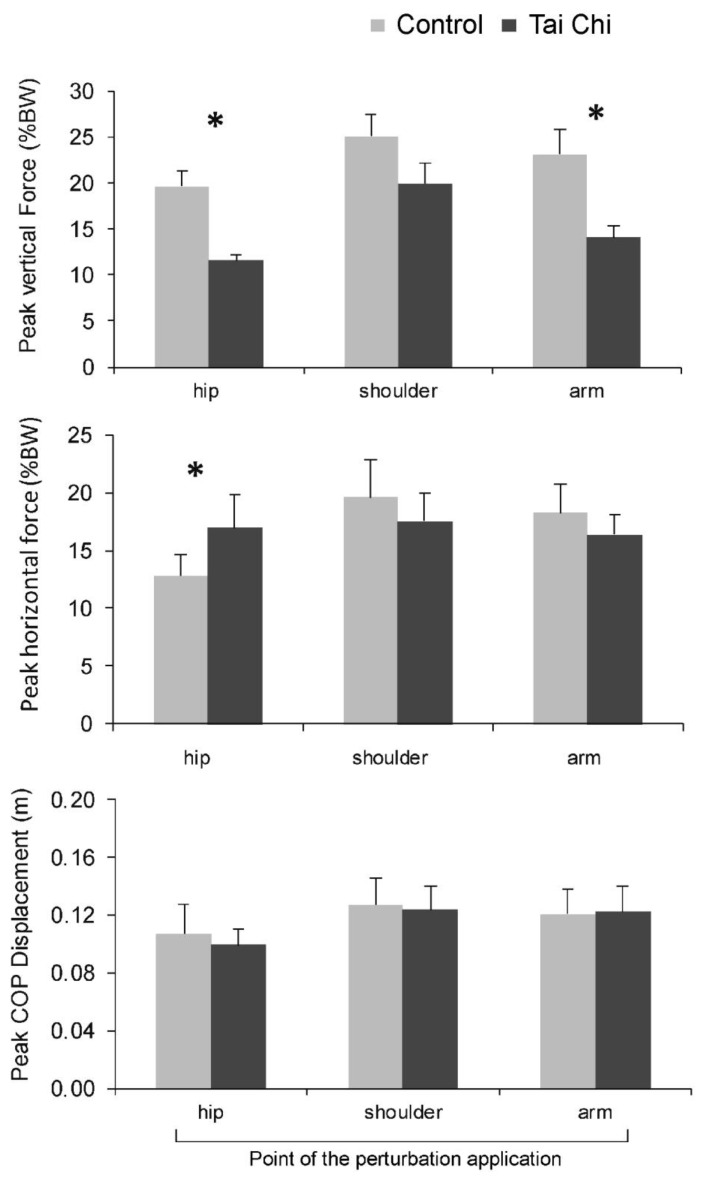
Peak vertical force (**top**), horizontal force (**middle**) and displacement of center of pressure (**bottom**) after sudden perturbation. * denotes statistically significant differences between the groups.

Examples of responses in terms of ground reaction forces and CoP displacement of two participants (one from each group) for the hip perturbation are shown in Figure 2. The main effect of group was statistically significant for F_z_ (F_(1,18)_ = 13.89, *p* = 0.002), but not for the maximal amplitude of F_y_ (F_(1,18)_ = 0.005, *p* = 0.943) or for maximal amplitudes of CoP (F_(1,18)_ = 0.124, *p* = 0.729) (Figure 3). Specifically, the Tai Chi group produced lower F_z_ amplitudes in response to perturbations at the hip (t = 0.044, *p* = 0.002, d = 1.86) and at the arms (t = 3.326, *p* = 0.005, d = 1.31), but not at the shoulder (t = 1.868, *p* = 0.078) (Figure 3, top). For the hip perturbation, the Tai Chi group produced larger F_y_ (t = 0.841; *p* < 0.001, d = 1.01). However, F_y_ was very similar between groups for shoulder and arm perturbations (t = 1.75–1.84; *p* = 0.082–0.099). Moreover, CoP displacement was not different between groups (t = 0.21–0.85; *p* = 0.410–830).

**Figure 4:**
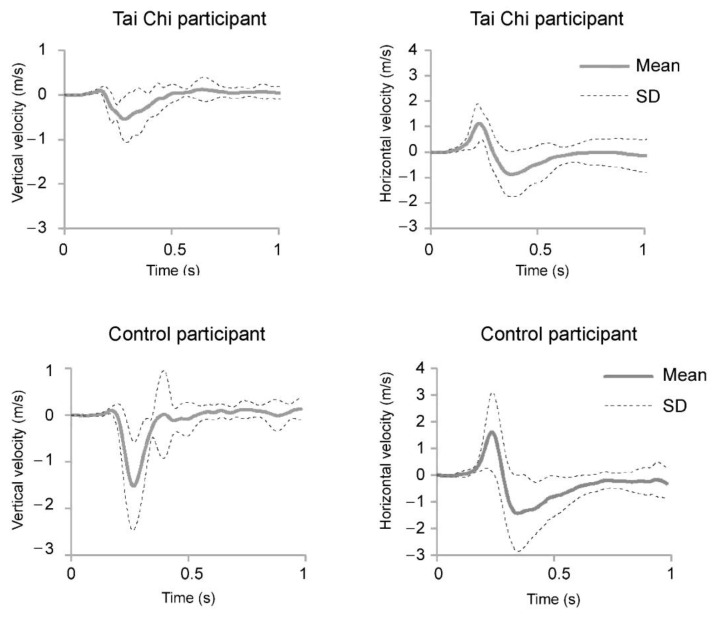
Example of vertical (**left**) and horizontal (**right**) knee velocity after hip perturbation.

Examples of responses to hip perturbation in terms of knee velocities (V_z_ and V_y_) are presented in Figure 4. There were statistically significant group effects for V_z_ at all three body sites—knee (F_(1,18)_ = 7.516, *p* = 0.013), hip (F_(1,18)_ = 5.949, *p* = 0.025), and shoulder (F_(1,18)_ = 5.359, *p* = 0.033). Post-hoc test showed statistically significant group differences for knee V_z_ after shoulder perturbation (t = 2.588, *p* = 0.026, d = 3.2), hip V_z_ after the hip perturbation (t = 3.138, *p* = 0.006, d = 2.71) and shoulder V_z_ after both hip perturbation (t = 2.219, *p* = 0.040, d = 1.87) and arm perturbation (t = 2.109, *p* = 0.049, d = 1.91).

**Figure 5:**
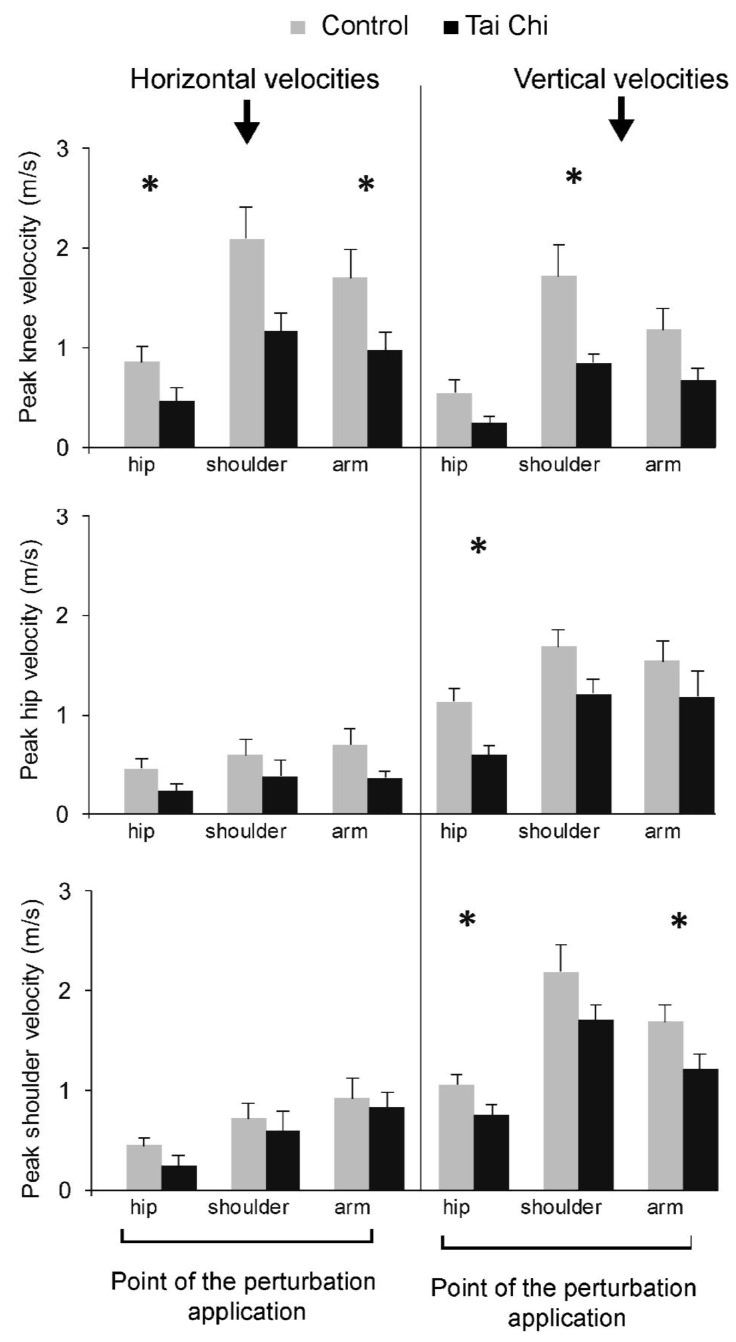
Peak horizontal (**left**) and vertical (**right**) velocities at the knee (**top**), hip (**middle**), and shoulder (**bottom**) joints for all three perturbation areas. * denotes statistically significant differences between the groups.

There were statistically significant main group effects for V_y_ at the knee (F_(1,18)_ = 10.438, *p* = 0.005), but not for shoulder or hip V_y_. (*p* = 0.342). Post-hoc tests for knee V_y_ showed statistically significant differences for shoulder (t = 2.512, *p* = 0.022, d = 2.05) and arm perturbations (t = 2.165, *p* = 0.047, d = 2.55) (Figure 5).

## 4. Discussion

In this study, we compared the reactive postural responses to horizontal unloading perturbations in Tai Chi practitioners and healthy adults. We designed an experimental setup that simulated the perturbations experienced during the “pushing hands” technique, which is common in Tai Chi. We hypothesized that Tai Chi participants will show superior reactive control of posture, which will be reflected in lower peak ground reaction forces, lower peak CoP displacement and lower peak body movement velocities after the perturbations. Although not all of the outcome measures showed statistically significant differences between the groups, the Tai Chi participants generally exhibited better reactive postural control, which confirms our hypothesis.

Previously, Tai Chi training has been shown to improve postural control during standing [1,2] and walking [3], as well as the ability to recover balance after a slip [3]. The present study indicated that it could also offer an effective approach to counteract the deterioration of reactive postural control, which is seen in older adults [25] and patients with neuromuscular diseases [20,26,27]. Importantly, previous studies have already demonstrated that performing Tai Chi is safe and effective for Parkinson’s disease [6] and multiple sclerosis [28] patients. Acute improvements of postural responses has been shown before in multiple sclerosis patients [29] and older adults with mild cognitive impairment [30], after single-session ball throwing exercise. Moreover, improvements in anticipatory postural control have also been noted with long-term exercise [31]. The results of our study suggest that long-term Tai Chi exercise could be effective in improving reactive postural control—in particular, the ability to cope with horizontal perturbations.

The differences between the groups were more common when examining responses to hip (5/9 outcome measures) and arm perturbations (3/9 outcome measures compared to shoulder perturbations (1/9 outcomes measures). This indicates the observed adaptations could be task-specific. Such phenomena have been reported by a systematic review of static balance studies [17]. In other words, the literature suggests that balance training improves the performance in trained tasks, but has only minor or no effects on nontrained tasks [17]. The perturbations applied below the hips are easier to cope with from a purely mechanical perspective (viewing the human body as an inverted pendulum), while introducing the arms to the experiment provided additional degrees of freedom which are beneficial for movement control [32]. Task-specificity of adaptations could also explain why previous studies using surface translation as a perturbation found no effects of Tai Chi training on reactive postural control [14].

According to the theories of postural control, the underlying mechanisms of adaptations observed in the present study could be the result of changes in intrinsic mechanical muscle stiffness, changes short-latency reflex responses (e.g., the early stretch reflex burst) or changes in longer-latency responses [33,34]. While previous studies have shown that muscle stiffness may be increased by strength training [35], it seem unlikely that this mechanism is responsible for the differences observed in our study. Namely, Tai Chi also involves movements that require large ranges of motion, which would be expected to have an opposite effect on muscle stiffness [36,37] and several authors have suggested that Tai Chi improves flexibility [3,4,5]. Moreover, the intrinsic mechanical stiffness is not among the largest contributors to the between-participant variance in postural responses [38]. Resistance training may increase the gain of short-latency reflex responses [39], although some studies found no such effect [40,41]. On the contrary, exercises requiring postural stability may even decrease these responses [39], which could be beneficial for static balance, as such adaptations may prevent excessive reflex-mediated joint oscillations [39,42]. It would be interesting to investigate whether there are contrasting effects of strength and postural control components within Tai Chi exercises. Regardless of this issue, further studies would be needed to determine the effects of Tai Chi training on short-latency responses.

The final possible explanations for the observed differences that Tai Chi practitioner had superior longer-latency feedback control, which involves the central nervous system. Interestingly, structural changes in the brain (more specifically, a thicker cortex in several areas) have been reported in Tai Chi practitioners [43]. However, it remains unknown whether these changes are in any way related to the reactive postural control. It could be that Tai Chi training improves the long-latency feedback control, either by changes in proprioceptive [44] or vestibular system [45], or possibly by improving the sensorimotor integration [34]. While a very complex study [33,38] would be needed to investigate these possibilities in detail, these mechanisms would also explain the improvements in static postural control that were documented after Tai Chi training [1,2,3]. This study did not provide any evidence into the underlying mechanisms of improved reactive postural control, but the findings are valuable from practical and clinical perspectives.

One of the limitations of this study is the lack of analyses of the response latencies, obscuring the contribution of latency and magnitude to the changes in reactive postural responses. This is important because both slower onset and smaller magnitudes of responses have been suggested as potential risk factors for falling in older adults [46] and possibly even for the low back pain [21]. Furthermore, the cross-sectional design utilized within the study does not allow definite conclusions regarding the cause-and-effect relationship. Interventional trials are needed in the future to verify our findings. As the present findings are most relevant for older adults and patients with neuromuscular diseases that affect postural control, a direct generalization of the results to these populations is not possible, and further studies are also needed for this view. Another important limitation of the study is the discrepancy between the groups regarding age. Thus, some confounding effect of age cannot be ruled out. Finally, the sample size in the present study was relatively small, and it could be that some of the existing differences were not statistically detected.

## 5. Conclusions

In conclusion, our study demonstrated that long-term Tai Chi training can improve the reactive postural responses after sudden perturbations in a horizontal direction. Hence, in addition to general health benefits, Tai Chi may represent an efficient approach to improve postural control. Further studies are needed to explain all of the underlying mechanisms of the positive effects of Tai Chi training. Nevertheless, Tai Chi training may be recommended for general population, older adults and patients with neuromuscular diseases in order to promote general health and well-being, or specific abilities, such as postural control.

## Figures and Tables

**Table 1:** Basic participant data.

	Tai Chi Group (*n* = 10)	Control Group (*n* = 10)
Male/Female	6/4	7/3
Age (years)	37.4 ± 7.8	28.8 ± 5.0
Height (cm)	176.2 ± 8.4	174.9 ± 7.2
Body mass (kg)	68.7 ± 10.9	72.0 ± 8.3
Body mass index (kg/m^2^)	22.1 ± 2.9	23.5 ± 1.5
Tai Chi experience (years)	7.0 ± 5.5	0

## Data Availability

The data are available upon request to corresponding author’s email.
